# Low force contractions induce fatigue consistent with muscle mRNA expression in people with spinal cord injury

**DOI:** 10.1002/phy2.248

**Published:** 2014-02-25

**Authors:** Michael A. Petrie, Manish Suneja, Elizabeth Faidley, Richard K. Shields

**Affiliations:** 1 Department of Physical Therapy and Rehabilitation Science, Carver College of Medicine, The University of Iowa, Iowa City, Iowa; 2 Department of Veterans Affairs, VA Medical Center, Iowa City, Iowa; 3 Department of Internal Medicine, Carver College of Medicine, The University of Iowa, Iowa City, Iowa

**Keywords:** Electrical stimulation, genotype, paralysis, phenotype, potentiation

## Abstract

Spinal cord injury (SCI) is associated with muscle atrophy, transformation of muscle fibers to a fast fatigable phenotype, metabolic inflexibility (diabetes), and neurogenic osteoporosis. Electrical stimulation of paralyzed muscle may mitigate muscle metabolic abnormalities after SCI, but there is a risk for a fracture to the osteoporotic skeletal system. The goal of this study was to determine if low force stimulation (3 Hz) causes fatigue of chronically paralyzed muscle consistent with selected muscle gene expression profiles. We tested 29 subjects, nine with a SCI and 20 without and SCI, during low force fatigue protocol. Three SCI and three non‐SCI subjects were muscle biopsied for gene and protein expression analysis. The fatigue index (FI) was 0.21 ± 0.27 and 0.91 ± 0.01 for the SCI and non‐SCI groups, respectively, supporting that the low force protocol physiologically fatigued the chronically paralyzed muscle. The post fatigue potentiation index (PI) for the SCI group was increased to 1.60 ± 0.06 (*P *<**0.001), while the non‐SCI group was 1.26 ± 0.02 supporting that calcium handling was compromised with the low force stimulation. The mRNA expression from genes that regulate atrophy and fast properties (MSTN, ANKRD1, MYH8, and MYCBP2) was up regulated, while genes that regulate oxidative and slow muscle properties (MYL3, SDHB, PDK2, and RyR1) were repressed in the chronic SCI muscle. MSTN, ANKRD1, MYH8, MYCBP2 gene expression was also repressed 3 h after the low force stimulation protocol. Taken together, these findings support that a low force single twitch activation protocol induces paralyzed muscle fatigue and subsequent gene regulation. These findings suggest that training with a low force protocol may elicit skeletal muscle adaptations in people with SCI.

## Abstract (precis)

e00248

People with spinal cord injury develop severe osteoporosis and diabetes because of decreased muscle activity. We developed a low force muscle activation protocol that challenges the muscle and is suitable to apply in those with bone demineralization. This is the first study that illustrates the magnitude of muscle fatigue and the associated molecular adaptations associated with key genes known to regulate proteins associated with muscle fatigue.

## Introduction

After spinal cord injury (SCI) the muscular system atrophies and the fibers lose oxidative capacity (Grimby et al. 1976; Shields 1995; Shields and Chang 1997; Shields et al. 1997). The skeletal system becomes osteoporotic so that fractures may occur even during simple passive functional tasks (Fattal et al. 2011). There is over 50% loss of muscle mass after SCI, which removes important stress to bone (Eser et al. 2004), but also compromises metabolism as approximately 75% of glucose uptake occurs in skeletal muscle (Bjornholm and Zierath 2005). Healthcare workers are challenged to safely and efficiently promote exercise/activity for people with chronic SCI who are known to have secondary musculoskeletal and metabolic instability (Dudley‐Javoroski and Shields 2006; LaVela et al. 2012).

Neuromuscular electrical stimulation (NMES) is one method used by clinicians to increase muscle activity in people with paralysis. The parameters selected during electrical stimulation are the current intensity, frequency, and pulse duration, which determine the force produced by skeletal muscle. When NMES is used to activate muscle of people with acute spinal cord injury (weeks to months after SCI), before musculoskeletal deterioration (osteoporosis), high intensity and high frequency stimulation is well tolerated (Shields et al. 1997, 2006a; Shields and Dudley‐Javoroski 2006, 2007). However, 1–2 years after SCI, the musculoskeletal system is compromised (Shields and Dudley‐Javoroski 2006; Dudley‐Javoroski and Shields 2008) and there may be risk of injury when stimulating paralyzed muscle with high forces (Hartkopp et al. 1998). Skeletal fractures currently are a leading public health risk to people with SCI (Krause et al. 2008; Heiden et al. 2013). Importantly, people with paralysis may have better adherence to regular exercise if it can be delivered anytime of the day from the wheelchair.

An effective strategy, from a feasibility and safety perspective, for people with chronic SCI, would be to offer a low force protocol that also challenges the paralyzed muscle as evident by muscle fatigue. If a low force strategy is accomplished by using lower stimulation intensities, then a portion of the muscle is never recruited, and, therefore, not adapted with training. Conversely, by using a high stimulation intensity strategy (>100–250 milliamps) with a low frequency (1–5 Hz), all muscle fibers can be recruited but at a single twitch force level. Most studies use stimulation frequencies that range from 15 to 50 Hertz (Burke et al. 1973; Stein et al. 1992; Shields 1995; Shields et al. 1997, 2006a), including one study that reported a fracture (Hartkopp et al. 1998). We located three studies that used a low force stimulation protocol (<5 Hz) for muscle training; however two of these studies were in animal models (Eisenberg and Gilai 1979; Metzger and Fitts 1987) and the only human case was on a single subject (Ryan et al. 2013).

The physiologic phenotype of skeletal muscle is commonly measured by the ability for a muscle to sustain a given force. Muscle fatigue, defined as the change in muscle force during repetitive activation, provides an index of the muscle's physiologic capacity. Muscles that have a mid‐ to high‐fatigue resistance are considered oxidative and capable of using carbohydrates and lipids for fuel during long duration activity (Martin et al. 1988; Callister et al. 2004); however, muscles that have a low fatigue resistance are glycolytic, primarily using anaerobic pathways and stored glycogen as fuel during short duration activity (Martin et al. 1988; Callister et al. 2004). Recently, fast, fatigable, glycolytic muscle has been linked to insulin receptor resistance and diabetes (Mootha et al. 2003; Palsgaard et al. 2009). However, there is a gap in the literature regarding the extent to which repetitive low force contractions can challenge the chronically fast fatigable paralyzed muscle by inducing fatigue.

Repetitive stimulation of paralyzed muscle with higher force trains (15–50 Hz) causes extensive long duration muscle fatigue (Shields and Chang 1997; Shields et al. 1997; Iguchi et al. 2008). The long duration fatigue (also called low frequency fatigue) is thought to be due to impaired Ca^2+^ release from the sarcoplasmic reticulum (Westerblad et al. 1993). A competing process is potentiation, whereby stimulating a muscle that was previously fatigued causes a “staircase phenomenon” or an incremental increase in force with continued activation (Rassier 2000; Rassier and Macintosh 2000). The mechanism for potentiation is believed to be due to increased sensitivity of Ca^2+^ to the actin‐myosin complex during subsequent twitches (Moore and Stull 1984). Several genes have been identified that play a prominent role in encoding proteins that control physiological responses during repetitive electrical stimulation. Specifically, mRNAs from genes for atrophy (MSTN) (Jones et al. 2004), fast‐twitch, fatigable muscle (ANKRD1, MYH8 and MYCBP2) (Qin et al. 1994; Nakamura et al. 2002; Stevenson et al. 2006), slow twitch contractile properties (MYL3) (Alapat et al. 2009; Meyer et al. 2013), mitochondrial oxidative metabolism (SDHB, PDK2) (Kita et al. 1990; Oh et al. 1996; Jeong et al. 2012), and excitation‐contraction coupling (RyR1) (MacIntosh et al. 2012) are known regulators of the muscle phenotype.

Our long term goal is to determine if a safe and feasible low frequency/low force muscle training protocol can induce long term metabolic, physiologic, and molecular adaptations in humans with chronic SCI, ultimately translating into improved overall systemic metabolic health. In this study, we sought to test if muscle fatigue can be induced with a repetitive single twitch, low force protocol and whether the physiologic phenotype is consistent with the underlying mRNA expression for select genes in people with and without chronic paralyzed muscle. We focus our molecular examination on select genes known to be associated with atrophy, fatigue, and fast glycolytic muscle phenotypes.

The purpose of this study was to compare the physiologic properties of chronically paralyzed and non‐paralyzed muscle (fatigue and potentiation) during repetitive activation using a low force generating stimulation protocol (3 Hz). Our secondary purpose was to examine if fatigability, induced via a low force stimulation protocol, is consistent with the molecular expression of genes linked to each groups’ muscle phenotype. We hypothesized that human chronically paralyzed muscle would be sensitive to a low frequency stimulation protocol consistent with the gene expression profile.

## Methods

### Subjects

Twenty nine people, nine men with a complete motor spinal cord injury (American Spinal Injury Association ASIA‐A) and 20 people without SCI (10 men and 10 women) participated in a low frequency stimulation protocol (Table 1). The nine subjects with SCI had increased tendon reflexes consistent with upper motor neuron lesions. Some of the SCI subjects previously received electrical muscle stimulation training; however, all SCI subjects had been discontinued any electrical stimulation for at least 1 year before participating in this study. Three subjects (subject 1, 3, and 6 with SCI) and three non‐SCI subjects were biopsied. One subject (subject 6) underwent a biopsy 3 h after a low force protocol. This study was approved by the University of Iowa Human Subjects Office institutional review board. All subjects provided written informed consent before participation.

**Table 1.** Subject characteristics.

Subject	Gender	Age	Injury level	Years post‐injury
SCI 1	M	49	T4	5
SCI 2	M	63	T12	4
SCI 3	M	19	T8	3
SCI 4	M	27	T8	7
SCI 5	M	45	C5	26
SCI 6	M	31	T10	5
SCI 7	M	22	C4‐6	1
SCI 8	M	33	T5	9
SCI 9	M	35	T9	13
NON 1	M	24	–	–
NON 2	F	24	–	–
NON 3	F	24	–	–
NON 4	M	26	–	–
NON 5	F	24	–	–
NON 6	F	24	–	–
NON 7	M	24	–	–
NON 8	M	24	–	–
NON 9	M	25	–	–
NON 10	M	25	–	–
NON 11	F	23	–	–
NON 12	F	23	–	–
NON 13	M	25	–	–
NON 14	F	23	–	–
NON 15	M	24	–	–
NON 16	M	25	–	–
NON 17	F	22	–	–
NON 18	M	23	–	–
NON 19	F	24	–	–
NON 20	F	24	–	–

M, men; F, women; C, cervical; T, thoracic.

Subject with and without spinal cord injury are denoted by SCI and NON, respectively.

### Physiological testing instrumentation

**Figure 1.**
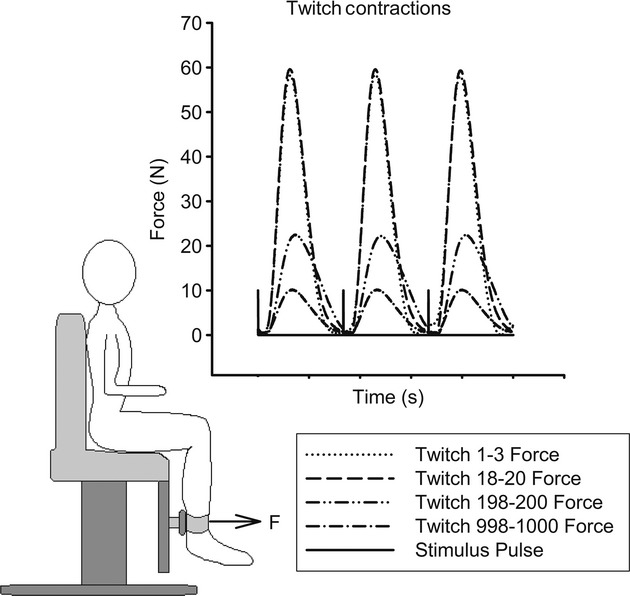
Torque measurement apparatus, with adjustable load cell and stabilization cuff (bottom). Representative twitches at the start, the peak, and the end of the first bout of the 3 Hz stimulation protocol in paralyzed muscle (top). The initial and peak twitches show little difference in amplitude, but the later twitches show a decrease in amplitude.

Subjects sat in a wheel chair or standard chair with the testing limb ankle secured to a force transducer. The testing limb thigh and knee angle were set to 90°. The transducer apparatus consisted of a padded, semicircular metal plate that cupped the posterior surface of the leg. This plate was connected to a force transducer (1500ASK0299, Interface, Scottsdale, AZ) that was mounted to a rigid metal plate. The transducer could be adjusted vertically to suit the height of the subject's medial malleolus. A padded strap secured the tibia to the force transducer apparatus (Fig. 1).

Self‐adhesive 7 cm × 13 cm oval carbon electrodes (EMPI, Inc. St. Paul, MN) were adhered to the skin over the quadriceps muscles, consistent with a previously reported protocol (Dudley‐Javoroski et al. 2008). In brief, the distal margin of the distal electrode was placed over the distal‐most palpable border of the vastus lateralis. The proximal electrode was positioned as close to the inguinal crease as possible, with the medial‐margin lateral to the adductor muscle group. Electrodes were connected via shielded cabling to a constant current electrical muscle stimulator unit (Digitimer model, DS7A, Digitimer, Welwyn Garden City, Hertfordshire, UK). Digital pulses controlled the stimulator from a data‐acquisition board (NI USB‐6221BNC, National Instruments, Austin, TX) under software control (Labview 2012, National Instruments, Austin, TX). The stimulator was set to deliver 200 *μ*s pulses at intensities up to 300 mA. All testing was conducted at intensities that elicited maximal twitch forces, verified by no increase in force with a subsequent increase in stimulation intensity. Our non‐SCI subjects were lean and achieved maximal contractions at around 100 mA. Because individuals with SCI typically have more resistance to stimulation (edema, subcutaneous fat), a higher current intensity was required to achieve the maximal contraction (~150 mA on average). The force and stimulator signals were amplified and sampled at a rate of 2000 Hz using a customized software application.

### Experimental protocol

Single stimulus pulses were delivered at progressively increasing intensities up to 300 mA for the SCI and up to 100 mA for the non‐SCI subjects to ensure supra maximal muscle stimulation. All stimulus pulses were widely spaced (10 sec between pulse) to minimize muscle fatigue before beginning the testing procedure. The testing procedure consisted of two bouts. The first bout of the testing procedure consisted of a single train of 1000 stimulus pulses delivered at 3 Hz. The second bout consisted of a train of 200 stimulus pulses delivered at 3 Hz. The second bout followed a 5‐min recovery period. Our preliminary testing indicated 1000 pulses at 3 Hz fatigued the muscle during bout 1. During bout 2, our preliminary studies indicated muscle potentiation was saturated by 200 pulses.

### Muscle physiological property analysis

We focused our analysis on two physiological variables consisting of the fatigue index (FI), and the potentiation index (PI). We also analyzed the change in the twitch speed properties by calculating a normalized rate of force development for each twitch. The FI was defined as the ratio of the final peak force to the peak twitch force within a bout. The PI was defined as the ratio of the peak twitch force and the initial twitch force. To determine the FI and PI, the peak twitch force was defined as the maximum peak amplitude of the force signal for all twitches within a bout. The normalized rate of force development was determined by calculating the slope between 20% and 80% of the peak twitch force and then dividing the slope by the peak twitch.

### Muscle biopsy, exon microarray procedure, protein analysis

The biopsy procedure has been previously described (Adams et al. 2011). Briefly, a percutaneous muscle biopsy was taken from the vastus lateralis muscle using a Temno biopsy needle (T1420, Cardinal Health, Dublin, OH) under ultrasound guidance within a sterile field. Several passes of the needle were used to collect a wide sampling range within the muscle. Following harvest, muscle biopsy samples were immediately placed in RNALater (Ambion) for RNA extraction or flash frozen in liquid nitrogen for protein extraction and stored at −86°C until further use.

RNA was extracted using the RNEasy Fibrous Tissue Kit (Qiagen). DNAse was included in the protocol to ensure absence of genomic DNA in final samples. RNA samples were eluted in water and quantified via a NanoDrop method. In addition, the quality of each sample was assayed using the Agilent 2100 Bioanalyzer (Agilent Technologies, Inc., Santa Clara, CA).

Microarray hybridizations were performed at the University of Iowa DNA Facility. Briefly, 50 ng RNA was converted to SPIA amplified cDNA using the WT‐Ovation Pico RNA Amplification System, v1 (NuGEN Technologies, San Carlos, CA, Cat. #3300) according to the manufacturer's recommended protocol. The amplified SPIA cDNA product was purified through a QIAGEN MinElute Reaction Cleanup column (QIAGEN Cat #28204) according to modifications from NuGEN. Four *μ*g of SPIA amplified DNA were used to generate ST‐cDNA using the WT‐Ovation Exon Module v1 (NuGEN Technologies, Cat #2000) and again cleaned up with the Qiagen column as above. 5 *μ*g of this product were fragmented (average fragment size = 85 bases) and biotin labeled using the NuGEN FL‐Ovation cDNA Biotin Module, v2 (NuGEN Technologies, Cat. #4200) per the manufacturer's recommendations. The resulting biotin‐labeled cDNA was mixed with Affymetrix eukaryotic hybridization buffer (Affymetrix, Inc., Santa Clara, CA), placed onto Human Exon 1.0 ST arrays (Part No. 900650), and incubated at 45°C for 18 h with 60 rpm rotation in an Affymetrix Model 640 Genechip Hybridization Oven. Following hybridization, the arrays were washed, stained with streptavidin‐phycoerythrin (Molecular Probes, Inc., Eugene, OR), signal amplified with antistreptavidin antibody (Vector Laboratories, Inc., Burlingame, CA) using the Affymetrix Model 450 Fluidics Station. Arrays were scanned with the Affymetrix Model 3000 scanner with 7G upgrade and data were collected using the GeneChip Operating Software (GCOS) v1.4 (Affymetrix Inc, Santa Clara, CA).

The Affymetrix Human Exon 1.0 ST arrays were initially normalized and summarized with a Robust Multi‐array Average (RMA) using Partek Genomic Suites (v6.6 © 2013 Partek, Inc., St. Louis, MO). Resulting exon expression intensities were further averaged to define the expression intensity for a specific gene. The SCI and non‐SCI signal intensities, relative to the mean signal intensity, were compared to identify expression similarities and differences. Expression intensities were verified using several housekeeping genes (REEP5, PSMB2, C1orf43, and CMP25A), all resulting in similar gene intensity levels. We also calculated the fold change for one subject who had the activated and the inactivated vastus lateralis biopsied 3 h following a low force protocol. The fold change was calculated as the ratio of the stimulated mRNA intensity/non‐stimulated mRNA intensity from the same person.

We analyzed the protein levels for two genes differentially expressed between the SCI and non‐SCI groups. Western blots were carried out to quantify protein concentrations for SERCA2 and SDHA. SERCA2 is associated with fatigue resistant skeletal muscle and SDHA is a subunit of a key enzyme in the citric acid cycle. The flash frozen biopsy samples were homogenized using a glass pestle in 1X PBS + 0.5% Triton X‐100 and Complete Protease Inhibitor Cocktail (Roche). Samples were vortexed thoroughly in the presence of detergent and agitated for 2 h at 4°C. Finally, samples were centrifuged twice at 16,300×g for 10 min each to remove insoluble matter. Resulting supernatants were assayed using the Bradford method. Samples were loaded in equal quantities, separated via electrophoresis on ready gels (Bio‐Rad) and blotted onto PVDF membranes. Membranes were blocked in 3% BSA in TBS + 0.1% Tween 20 (TBSTw) for 1 h, then incubated at 4°C overnight in SDHA or SERCA2 (both from Cell Signaling, Danvers, MS), each diluted to 1:1000 in block solution. Three rinses in TBSTw were performed before 1 h incubation in HRP‐conjugated anti‐rabbit secondary antibody (GE Healthcare Life Sciences) diluted to 1:10,000 in block solution. Finally, membranes were rinsed three times in TBStw; protein standards and secondary antibodies were visualized using streptactin‐HRP (Bio‐Rad). Chemiluminescence results were developed using the ImmunStar WesternC Substrate (Bio‐Rad) and results were quantified using the ChemiDoc XRS system and Quantity One software (Bio‐Rad) and normalized to subject's GAPDH level.

### Statistical analysis

A two‐way split plot ANOVA with repeated measures was used to compare the FI and PI between the SCI and non‐SCI cohorts (Subject × Bout × FI or PI). We carried out *post hoc* testing using the Tukey testing procedure, which accounts for experimental type I errors for all pair wise comparisons. SCI subjects were tested bilaterally, providing a replicate sample that was included in the statistical model; however, the non‐SCI subjects were unilaterally tested and did not have replicate samples. All statistical significance was unchanged, regardless of whether the replicate samples were included as independent samples. Gene and protein expression levels were tested using an independent t‐test. A significance level of (*P *<**0.05) was used for all testing procedures.

## Results

### Physiologic response to the low force protocol

**Figure 2.**
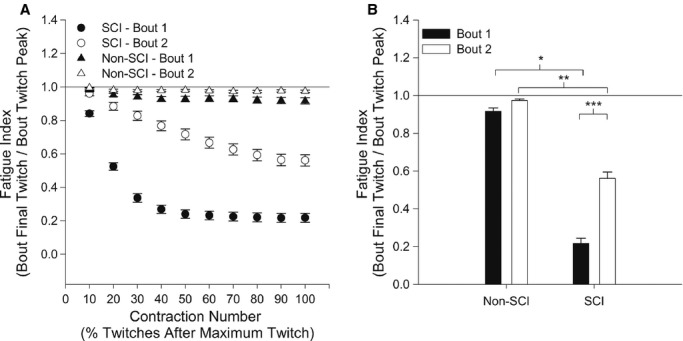
(A)The group means and standard errors for the fatigue index (FI), as a function of the maximum twitch, for bout 1 and bout 2. The twitches after the maximum twitch within each bout were grouped in bins of 10% of the remaining twitches. (B) The mean fatigue index (FI) for each group for bout 1 and bout 2. *Significant difference between SCI and non‐SCI for bout 1; **Significant difference between SCI and non‐SCI for bout 2; ***Significant difference between bout 1 and 2 for the SCI group.

The SCI max twitch force decreased from bout 1 to bout 2 by over 40% (46.55 ± 27.88N to26.72 ± 17.03N). Conversely, the non‐SCI max twitch force decreased from bout 1 to bout 2 by only 15% (62.29 ± 20.31N to 52.88 ± 15.66). The paralyzed muscle was highly fatigable to the low force stimulation protocol while the non‐paralyzed muscle was not fatigable. The fatigue index (FI) was 0.21 ± 0.27 and 0.91 ± 0.01 for the SCI and non‐SCI groups, respectively, at the end of bout 1 (Fig. 2A,B). After bout 2, the SCI group FI was 0.56 ± 0.03 (*P *<**0.001) and the non‐SCI group remained at 0.97 ± 0.007. The FI was ~80% and 50% lower for the SCI group when compared to the non‐SCI group for bout 1 (*P *<**0.001) and bout 2 (*P *<**0.001), respectively (Fig. 2A,B). The normalized twitch rate of rise for the non‐SCI group was unchanged from twitch 10 to twitch 1000 (12.8 sec^−1^ to 12.5 sec^−1^). Conversely, the normalized rate of rise was faster at the start (15.0 sec^−1^) but showed significant slowing to 11.1 sec^−1^ by the 1000th contraction for the SCI group. Because of the extensive fatigue, there were no measureable effects in bout 2 for the speed properties.

**Figure 3.**
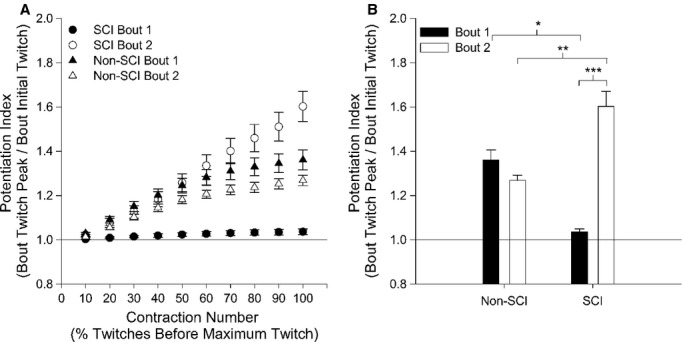
(A) The group means and standard errors for the potentiation index (PI), as a function of the maximum twitch, for bout 1 and bout 2. The twitches before the maximum twitch within each bout were grouped in bins of 10% of the preceding twitches. (B) The potentiation index (PI) for each group for bout 1 and 2. *Significant difference between SCI and non‐SCI for bout 1; **Significant difference between SCI and non‐SCI for bout 2; ***Significant difference between bout 1 and 2 for the SCI.

The chronic paralyzed muscle also showed a progressive increase in peak force during repetitive activation, 5 min after the muscle was fatigued. The potentiation index (PI) for the SCI group was unchanged in bout 1(1.04 ± 0.01), while the non‐SCI cohort was increased to 1.36 ± 0.04 in bout 1 (Fig. 3A,B). However, in bout 2, the PI for SCI increased to 1.60 ± 0.06 (*P *<**0.001), while the non‐SCI group decreased to 1.26 ± 0.02 (*P *=**0.08). The PI was significantly increased for the SCI group during bout 2 (*P *<**0.001), when the muscle was in the fatigued state (Fig. 3A,B).

### Atrophy and fast fatigable gene expression

**Figure 4.**
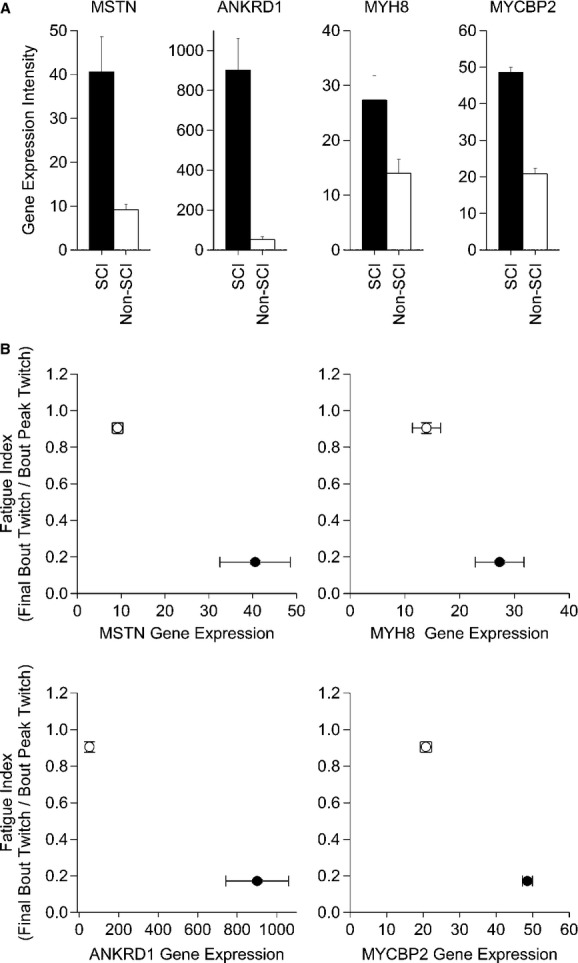
(A) The relative gene expression intensity for MSTN, ANKRD1, MYH8, MYCBP2 between a chronic SCI and non‐SCI muscle phenotype. (B) A bidirectional standard error plot of the fatigue index versus the relative gene expression intensity (MSTN, ANKRD1, MYH8, and MYCBP2) for the chronic SCI phenotype (closed circle) and non‐SCI phenotype (open circle). The relative expression intensity for all genes were significant at the *P *<**0.05 level (see text for details).

Both the FI and PI indicated that the low force stimulation, induced by a low frequency stimulation protocol, differentiated the SCI and non‐SCI phenotype. We next examined key mRNAs from genes known to regulate the skeletal muscle phenotype. We focused this analysis on four genes that are closely linked to muscle atrophy (MSTN) (Jones et al. 2004) and fast‐twitch, glycolytic muscle properties (ANKRD1, MYH8 and MYCBP2) (Qin et al. 1994; Nakamura et al. 2002; Stevenson et al. 2006). The mRNAs for genes MSTN, ANKRD1, MYH8 and MYCBP2 were all up regulated in the SCI group (*P *<**0.003, 0.001, 0.026, and 0.001), respectively, consistent with an atrophied muscle made up of fast glycolytic fibers (Fig. 4A). A plot of the FI, relative to the mRNAs expression levels, indicated that the low FI was consistent with the up regulation of these selected genes relative to the non‐SCI group (Fig. 4B).

### Oxidative metabolism and E‐C coupling gene expression

We examined four mRNAs for genes known to regulate slow contractile properties (MYL3) (Alapat et al. 2009; Meyer et al. 2013), oxidative metabolism (SDHB, PDK2) (Kita et al. 1990; Jeong et al. 2012), and excitation contraction coupling (RyR1) (MacIntosh et al. 2012).

**Figure 5.**
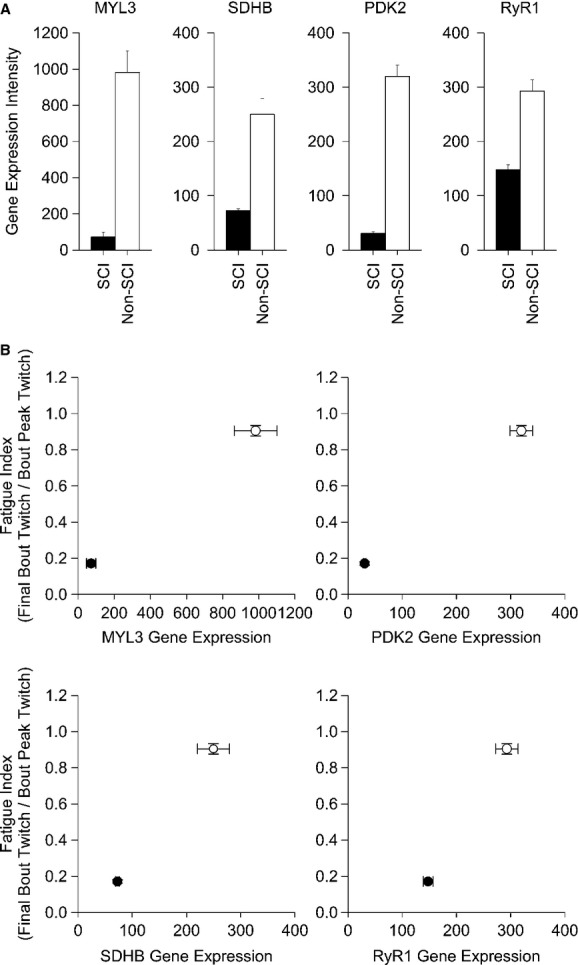
(A) The relative gene expression intensity for MSTN, ANKRD1, MYH8, MYCBP2 between a chronic SCI and non‐SCI muscle phenotype. (B) A bidirectional standard error plot of the fatigue index versus the relative gene expression intensity (MYL3, SDHB, PDK2, and RYR1) for the chronic SCI phenotype (closed circle) and non‐SCI phenotype (open circle). The relative expression intensity for all genes was significant at the *P *<**0.05 level (see text for details).

The mRNAs for genes MYL3, SDHB, PDK2, and RyR1 were all significantly repressed in the chronic SCI groups when compared to the non‐SCI group (*P *<**0.001, 0.002, 0.012, and 0.018), respectively (Fig. 5A). These findings are consistent with muscle fibers that show rapid fatigue and post activation potentiation. A plot of the FI, relative to the gene expression levels, indicated that the low FI was consistent with the down regulation of these selected genes relative to the non‐SCI group (Fig. 5B).

### Muscle protein analysis

**Figure 6.**
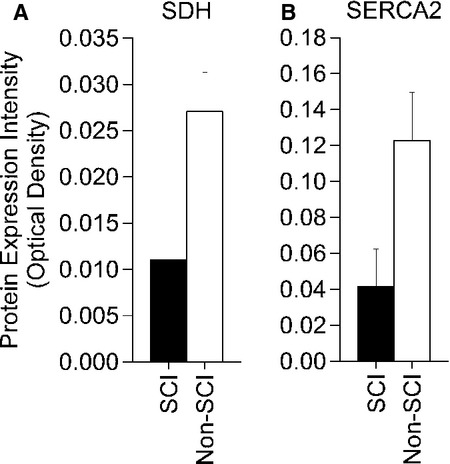
The relative protein expression level of SDH, an oxidative enzyme in the citric acid cycle which is partially translated by SDHB, was significantly depressed in chronic SCI compared to non‐SCI at the *P *<**0.05 level(A). The relative protein expression level of SERCA2, a calcium transport protein associated with slow‐twitch muscle and the associated RyR1, was significantly depressed in chronic SCI compared to non‐SCI at the *P *<**0.10 level (B).

We examined two proteins associated with muscle fatigue (oxidative metabolism) and excitation‐contraction coupling calcium kinetics. Succinate dehydrogenase (SDH), a mitochondrial oxidative enzyme, was significantly reduced in the chronic SCI group (*P *=**0.021, Fig. 6A). Sarcoplasmic/endoplasmic reticulum Ca^2+^‐ATPase (SERCA2), a key protein facilitating calcium uptake during repetitive activation of paralyzed muscle, was also reduced in the SCI group (*P *=**0.07) (Fig. 6B).

### Acute gene expression response

**Figure 7.**
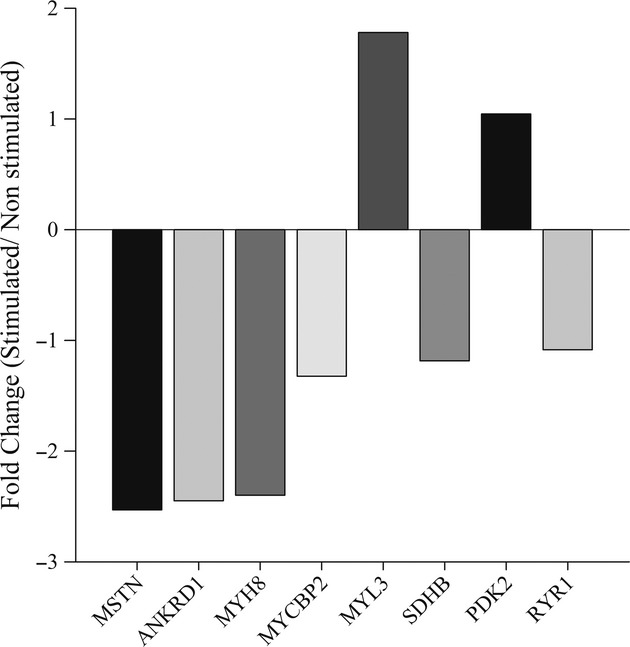
The gene expression changes from a single subject (subject 6) 3 h after a low force stimulation protocol. The expression of MSTN, ANKRD1, MYH8, MYCBP2, MYL3, SDHB, PDK2, and RYR1 is shown as the fold change difference between the stimulated muscle relative to the non stimulated muscle.

Three hours following the low force protocol the mRNAs for MSTN, ANKRD1, MYH8 and MYCBP2 genes, associated with atrophy and glycolytic pathways, were repressed 150%, 140%, 130%, and 30%, respectively (Fig. 7). The mRNAs for the MYL3 gene, associated with slow contractile properties was up regulated over 70%, while SDHB, PDK2, and RyR1 mRNAs were minimally altered 3 h after the exercise.

## Discussion

The objective of this study was to determine if a low‐force, repetitive activation protocol would induce fatigue and post fatigue potentiation. We discovered that 1000 stimulus pulses (twitches), delivered at a 3 Hz stimulation frequency, caused fatigue of the paralyzed quadriceps muscle in people with long term paralysis but did not fatigue the quadriceps of people without paralysis. The fatigability responses were consistent with each group's gene expression signatures. These findings support that a single twitch, low force protocol, induced fatigue of the chronically paralyzed muscle while minimizing deleterious stress to the underlying skeletal system. Based on these findings, this protocol may offer a safe strategy to fatigue the chronically paralyzed muscle and regulate gene expression to enhance metabolic and physiological adaptations.

### Muscle inactivity and metabolic health

We previously described the physiological and histochemical properties of acute and chronic paralyzed muscle (Shields 1995; Shields and Dudley‐Javoroski 2006, 2007), and developed a method to train acutely paralyzed muscle, to prevent bone loss in people with acute SCI (Shields and Dudley‐Javoroski 2006, 2007; Dudley‐Javoroski et al. 2012; Dudley‐Javoroski and Shields 2013). However, individuals with chronic paralysis, who already have severe osteoporosis, may be limited in their capacity to train their paralyzed muscles because of fracture risk (Hartkopp et al. 1998). A protocol that minimizes force, but fatigues the muscle metabolic machinery, as examined in this study, may offer an alternative strategy to improve the health of chronically paralyzed muscle.

The link between muscle metabolic stability and fast glycolytic properties is now readily acknowledged. It has been shown that people with decreased insulin receptor sensitivity (diabetes) have a higher prominence of transformed fast glycolytic fatigable muscle fibers (Mootha et al. 2003; Palsgaard et al. 2009). The notion that skeletal muscle may be a key endocrine organ (Pedersen 2013) suggests that the systemic metabolic instability that is endemic to people with SCI (Lavela et al. 2006) is driven in part by the nearly 100% transformation of slow oxidative muscle fibers to fast fatigable muscle fibers (Shields 1995). A stimulation strategy to deliver repetitive activity to paralyzed muscle in an effort to down regulate glycolytic pathways and up regulate oxidative pathways, may improve muscle metabolism in people with SCI. In this study, we verified that a low force protocol induced muscle fatigue and post fatigue potentiation. In addition, 3 h after the low force protocol, several of the gene expression profiles moved in a direction consistent with non‐SCI muscle. Taken together, the delivery of this low force protocol, on a regular basis, and perhaps throughout the day, may induce adaptations that move fast glycolytic SCI muscle towards that of non‐SCI muscle.

New research in healthy people emphasize that sitting is an independent risk factor for metabolic disease (diabetes) (Healy et al. 2008; Chau et al. 2014). Episodic physical exercise in non paralyzed people does not mitigate the effects of sitting (Healy et al. 2008; Chau et al. 2014). This finding underscores the inherent problem for people with SCI, who sit most of their life. Even 1 h of physical activity per day did not compensate for the negative effects of excessive sitting on insulin sensitivity and plasma lipids in people without SCI (Duvivier et al. 2013). However, “minimal intensity” physical activity, distributed across the day for a longer duration, was most effective at reducing insulin levels during glucose tolerance tests (Duvivier et al. 2013). The low force generating 3 Hz protocol tested in this study, by virtue of the low risk to the skeletal system, may be tolerated several times a day to enhance glucose utilization in people with chronic paralysis and trigger a more stable systemic metabolic use of glucose. Importantly, this protocol may have high translatability because of its ease of use at home and from the wheelchair. Recently, Carty et al. reported improvements in aerobic capacity and body composition using sub tetanic muscle contractions in humans with SCI (Carty et al. 2012, 2013), which supports the notion that low force muscle exercise may induce systemic cardiovascular adaptations.

### High stimulation intensity with low frequency/force contractions

Much of what we know about muscle adaptations originates from volitional studies (Rivera‐Brown and Frontera 2012; Schoenfeld 2013). Because of the size principle, it is unnatural (or virtually impossible) to volitionally recruit the entire muscle at a low force during isometric contractions (Henneman et al. 1965). In order to volitionally increase muscle force, we are required to recruit more motor units. The protocol used in this study recruited the entire muscle, but at a frequency that would not allow the twitches to be summated. This raises the question, what role does peak force play in fatigue and mechano‐transduction in modulating gene expression? In this study, we observed an 88% (only 9% loss in non‐SCI) loss of force after 1000 stimulus pulses delivered at 3 Hz in chronic paralyzed muscle. Using a modified Burke Fatigue Protocol (20 Hz; 333 ms on; 667 ms off; 120 contractions), we previously induced an 83% loss of force in chronically paralyzed muscle, despite generating an initial force that was 300% higher than the force used in this study (Shields 1995). Accordingly, it appears that muscle fatigue, induced by electrical stimulation of paralyzed muscle, may be independent of the peak force. When we examine the number of pulses delivered in several studies, we observe that most muscle fatigue occurs after approximately 500 stimulation pulses (Shields 1995). The principle that the number of stimulation pulses, and not force, determines the magnitude of fatigue in skeletal muscle was first proposed in 1983 (Marsden et al. 1983).

### Mechanisms contributing to fatigue

The activation of a muscle fiber is a complex process and failure may occur at various sites during electrical stimulation. NMES triggers nerve activation that elicits acetylcholine (ACH) release to elicit an action potential over the sarcolemma, which propagates to the transverse tubules (T‐tubules). The ryanodine (RyR) and dihydropyridine (DHPR) receptors sense the sarcolemmal action potential in the T‐tubules and release calcium (Ca^2+^) from the sarcoplasmic reticulum (SR). The Ca^2+^ influx interacts with troponin, freeing actin to bind with myosin and generate force. Muscle relaxation requires sarcoplasmic Ca^2+^ to be actively sequestered back in the SR against a diffusion gradient (active process). A calcium‐activated adenosine triphosphatase enzyme (SERCA) actively pumps Ca^2+^ into the SR, allowing troponin to inhibit actin‐myosin interactions. Adenosine triphosphate (ATP), the energy currency in the cell, is phosphorylated to adenosine diphosphate (ADP) and ultimately adenosine monophosphate (AMP, and inorganic phosphorous (P_i_). SERCA phosphorylates ATP to sequester Ca^2+^ during muscle relaxation, while myosin ATPase regulates the rate that cross‐bridges are cycled. A single stimulus pulse puts the muscle into an “active state” followed immediately by the “inactive state to return to the resting condition. When stimuli are repeated at a higher stimulation frequency (15–30 Hz), the sarcoplasmic Ca^2+^ concentration remains elevated, causing the twitches to summate and reducing the need to sequester Ca^2+^. Because muscle relaxation requires energy (active uptake of calcium), the protocol in this study induced complete relaxation 1000 times, a factor that may be important in challenging the paralyzed muscle.

We previously demonstrated that changes in M‐waves during fatigue of paralyzed muscle was independent of the change in muscle force (Shields 1995; Shields et al. 1997, 1998; Chang and Shields 2002). Hence, we do not believe that this single twitch protocol induced fatigue by compromising the neuromuscular transmission system. Thus, several underlying mechanisms including Ca^2+^ transient dysregulation, myosin light chain phosphorylation, and the accumulation of metabolic products may all have contributed to the fatigue observed in this study (Eisenberg and Gilai 1979; Duchateau et al. 1987; Fauler et al. 2012; MacIntosh et al. 2012). It is likely that the repeated muscle twitches triggered the buildup of byproducts of ATP phosphorylation (AMP and P_i_), which decreased the RyR sensitivity and the subsequent release of Ca^2+^ (Eisenberg and Gilai 1979; Duchateau et al. 1987; Fauler et al. 2012; MacIntosh et al. 2012). Given the robust difference in the RyR1 gene expression between the SCI and non‐SCI muscle, we reason that paralyzed muscle has a limited capacity to repetitively induce the active and resting states of the muscle during a single twitch. The capacity for substrates like glucose and fatty acids to be used as an energy source (oxidative pathways) to decrease the rate of byproduct (AMP and P) production is unknown, but clearly regulated by enzymes under molecular control (MYL3, SDHB, and PDK2). Consistent with our mRNA studies, these genes are significantly down regulated in the chronically paralyzed muscle. The mitochondria are responsible for aerobic metabolism and are the major contributor to ATP production in fatigue‐resistant muscle (Jacobs et al. 2013). Inactive muscle adapts by decreasing muscle size and mitochondrial number (Zierath and Hawley 2004; Stephenson and Hawley 2013). We recently demonstrated, in individuals with acute SCI, that higher force training protocols (15 Hz) up regulate several genes that promote mitochondrial enzymes and glucose utilization (Adams et al. 2011). Interestingly, 3 h after the low force stimulation protocol in this study, all four genes that regulate atrophy and glycolytic properties were down regulated, while one gene that regulates oxidative properties was up regulated. Although the long term training studies using a low frequency protocol have not been undertaken, we hypothesize, based on the responses from this study, that long term adaptations are possible. Importantly, these adaptations are detected early by this molecular analysis, and may be an important strategy to optimize the stimulation dose to enhance the muscle health of people with chronic SCI.

### Mechanisms contributing to post fatigue potentiation

Muscle force output may be modulated by the interaction between potentiation and fatigue (Rassier 2000; Rassier and Macintosh 2000). Activating a rested muscle phosphorylates myosin regulatory light chains (RLC), increasing sensitivity to Ca^2+^ (Moore and Stull 1984; Tubman et al. 1996, 1997; Vandenboom and Houston 1996; MacIntosh et al. 2012). The increased sensitivity leads to more actin‐myosin interactions and increase the total force output (Moore and Stull 1984; Tubman et al. 1996, 1997; Vandenboom and Houston 1996; MacIntosh et al. 2012). Continual activation of the muscle phosphorylates RyR, decreasing Ca^2+^ release, mitigating the increased force obtained from RLC phosphorylation. Our findings were consistent with the notion that long duration fatigue compromises the rate of phosphorylation of chronically paralyzed muscle.

## Summary and Conclusions

This study demonstrated that a low force single twitch stimulation protocol fatigued the human chronically paralyzed muscle. The fatigue index, the potentiation index, and speed properties responded in a manner consistent with the mRNAs expressed for several genes known to regulate the physiological response to repetitive stimulation. A single bout of this low force protocol promoted the mRNAs of genes in a direction consistent with the non‐SCI subjects. Because individuals with SCI develop (1) severe osteoporosis, (2) have limited muscle activity, (3) impaired muscle glucose utilization, and (4) diabetes, this low force activation protocol may present a novel strategy to mitigate the incidence of metabolic disease in a convenient and safe manner. How training with a low force protocol affects muscle is unknown. Future studies are necessary to determine the longitudinal value of regular low force exercise in comparison to other methods to sustain the overall health of people with SCI.

## Acknowledgments

We thank Tom Bair of the University of Iowa DNA Facility for his expertise with the microarray analysis. We thank Colleen McHenry, Brandon Campbell, Shauna Dudley‐Javoroski, Ann Lawler, and Amy Kimball for assistance with the collection of data and development of the manuscript.

## Conflict of interest

The authors report no conflicts of interest.
